# Deep Reinforcement Learning–Based Online One-to-Multiple Charging Scheme in Wireless Rechargeable Sensor Network

**DOI:** 10.3390/s23083903

**Published:** 2023-04-12

**Authors:** Zheng Gong, Hao Wu, Yong Feng, Nianbo Liu

**Affiliations:** 1 Yunnan Key Laboratory of Computer Technology Applications, Kunming University of Science and Technology, Kunming 650500, China; 2 School of Computer Science and Engineering, University of Electronic Science and Technology of China, Chengdu 611731, China

**Keywords:** wireless rechargeable sensor network, one-to-multiple charging, charging sequence, charging amount, Double Dueling DQN

## Abstract

Wireless rechargeable sensor networks (WRSN) have been emerging as an effective solution to the energy constraint problem of wireless sensor networks (WSN). However, most of the existing charging schemes use Mobile Charging (MC) to charge nodes one-to-one and do not optimize MC scheduling from a more comprehensive perspective, leading to difficulties in meeting the huge energy demand of large-scale WSNs; therefore, one-to-multiple charging which can charge multiple nodes simultaneously may be a more reasonable choice. To achieve timely and efficient energy replenishment for large-scale WSN, we propose an online one-to-multiple charging scheme based on Deep Reinforcement Learning, which utilizes Double Dueling DQN (3DQN) to jointly optimize the scheduling of both the charging sequence of MC and the charging amount of nodes. The scheme cellularizes the whole network based on the effective charging distance of MC and uses 3DQN to determine the optimal charging cell sequence with the objective of minimizing dead nodes and adjusting the charging amount of each cell being recharged according to the nodes’ energy demand in the cell, the network survival time, and MC’s residual energy. To obtain better performance and timeliness to adapt to the varying environments, our scheme further utilizes Dueling DQN to improve the stability of training and uses Double DQN to reduce overestimation. Extensive simulation experiments show that our proposed scheme achieves better charging performance compared with several existing typical works, and it has significant advantages in terms of reducing node dead ratio and charging latency.

## 1. Introduction

Wireless sensor networks (WSN) have gradually served as an essential infrastructure and have been widely used in modern defense, national health, intelligent transportation, environmental monitoring, and so on [1,2]. For a long time, however, the limited battery energy of sensor nodes has been a key factor in restricting the performance and popularization of WSN. Benefiting form the breakthrough of wireless charging technology, a novel and practical solution is to deploy charging systems for WSN to form wireless rechargeable sensor networks (WRSN) [3], which consist of one or more mobile chargers (MC) with high-capacity batteries, a base station (B), a service station (S) that can recharge MC, and lots of sensor nodes. The base station and the service station are usually at the same location, collectively known as BS. MC can move autonomously and is equipped with a wireless charging device to replenish energy for nodes by wireless charging to reduce the number of dead nodes.

Since the death of nodes in WSN may lead to serious consequences such as link disconnection, how to optimize the scheduling of MC to reduce the number of node deaths has become critical and has attracted much attention from the research community. Many studies have been conducted to explore the charging schemes for WRSN, which can be divided into two main categories: offline schemes [4,5,6,7,8,9] and online schemes [10,11,12,13,14,15,16,17]. In the offline schemes, it is assumed that the energy consumption rates of sensor nodes are fixed or regular, and thus, MC can plan its charging path in advance and moves periodically along the planned path to replenish energy for the sensor nodes. Offline schemes usually study a fixed charging path that minimizes MC travel distance while minimizing the number of dead nodes. For example, work of [5] is to calculate a charging path that minimizes node mortality while reducing MC travel distance. However, due to WRSNs usually being deployed in close contact with the environment, the energy consumption of sensor nodes is dynamic and diverse in practice, affected by the surrounding environment, which shows that the pre-defined charging scheduling in the offline schemes cannot well adapt to the dynamicity of the surroundings, leading to a lack of flexibility and to inefficiency.

Unlike the offline schemes, in online schemes, each sensor node monitors its residual energy and proactively sends a charging request to MC once it falls below a threshold. MC will make real-time scheduling to choose a charging candidate node among the receiving requests. Therefore, due to the different request times of charging nodes, the number of request nodes is different, and a fixed charging path cannot be calculated for online charging scheme. Therefore, the optimization objective of the online charging scheme is not the charging path, but the charging sequence. Since the online charging schemes are closely linked to the environment, online charging schemes can be well adapted to the changing energy demand of the WRSN, which can further reduce node dead ratio, but the existing online works still have performance problems for the following reasons. Firstly, most of them do not take the optimization of the charging path into account in whole, which could bring about unnecessary movement of MC and thus increase charging cost. For example, the classical online charging scheme NJNP [18] only considers the spatial position relationship between nodes and MC, and ignores the influence of node remaining power.

**Figure 1:**
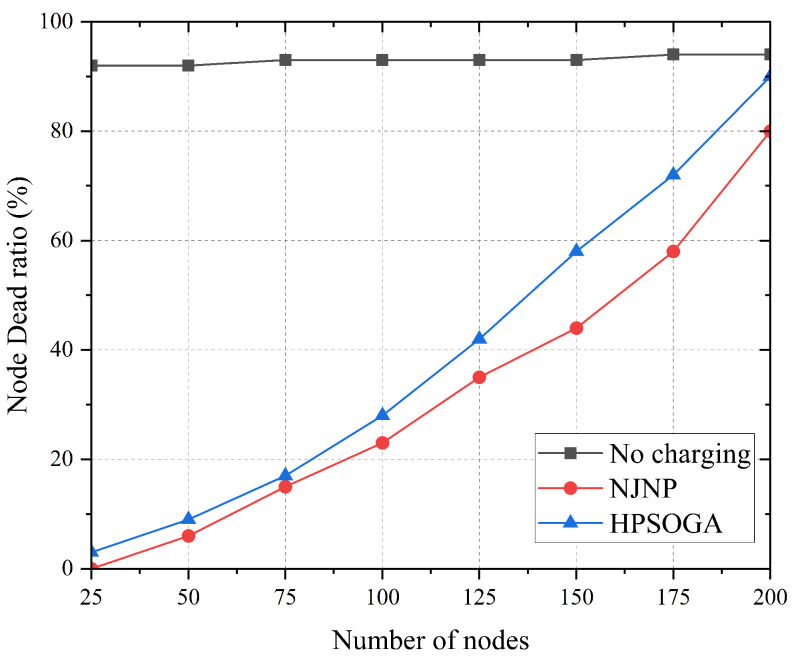
Impact of the number of nodes to the performance of the one-to-one charging scheme.

Secondly, most existing online schemes are based on one-to-one charging, by which MC can only charge one sensor at a time, making it prone to resulting in node failure for too long waiting time for charging, and is thus only suitable for small-scale WRSN. Figure 1 depicts the performance under the charging schemes HPSOGA [19] and NJNP using one-to-one charging scheme. As shown in Figure 1, HPSOGA and NJNP significantly reduce the node dead ratio when the number of nodes is small; however, its performance decreases severely as the network size increases. When the number of network nodes exceeds 100, the node dead ratio increases significantly and HPSOGA cannot effectively reduce the node dead ratio.

Thirdly, the existing online charging schemes lack explicitly analyzing and optimizing the charging amount that MC transfers to the sensor node(s) in each charging task. The charging amount is an important factor in determining the performance of the charging scheme; specifically, too much charging amount can lead to a long charging time, causing uncharged nodes to die before they are charged, while too little charging amount can make it difficult for nodes to run efficiently for a long time.

Aiming at the above problems, this paper proposes a novel solution. First of all, one-to-multiple charging [20], i.e., MC can charge multiple sensor nodes simultaneously, is adopted to increase the rate of energy replenishment of MC. Then, a new scheduling scheme is proposed to optimize both the charging sequence and the charging amount to improve performance. Classical optimization methods [21,22] are usually difficult to converge when tackling the above optimization problem with the large dimensionality of dynamic environment state, while reinforcement learning (RL) [23] uses a trial-and-error approach that allows agents to continuously learn as they interact with the environment in order to achieve a specific goal.

In classical RL process, the agent perceives state *s* from the environment and then selects action *a* based on state *s* and its own parameters; after performing action *a*, the environment state transitions from state *s* to the next state $s^{\prime }$ and provides a reward value $r^{\prime }$. The intelligent body learns between state transitions in the form of a sequence $\left(s,a,r^{\prime },s^{\prime }\right)$ and continuously optimizes its own parameters. Thus, RL has adaptability to dynamic environments and powerful decision-making capabilities, and is thus more suitable for optimizing MC scheduling. For example, the work of [15] uses reinforcement learning Q-learning, which combines RL and MC to reduce node mortality while increasing MC autonomy. However, the work of [15] is an offline charging scheme, which is difficult to apply in a dynamic WRSN environment.

Classical reinforcement learning, such as Q-learning, is only applicable to problems with limited state space and action space; it requires a data approximation function approach to deploy value functions and perform state updates, and requires manual design of high-quality learning features. In WRSN, with the large variety and number of sensor nodes and the large amount of continuous high-dimensional data such as residual energy, RL is difficult to apply to such problems. Deep learning [24], with its rapid development, has more powerful processing ability for continuous high-dimensional data. Therefore, reinforcement learning can be combined with deep learning to form deep reinforcement learning (DRL) with both high-dimensional continuous data processing ability and powerful decision-making ability. Therefore, DRL utilizes deep learning for high-dimensional continuous data processing and reinforcement learning for real-time decision making, which can well solve the problem of optimizing MC for scheduling in WRSN.

Specifically, in this paper, we propose an online one-to-multiple (OTM) charging scheme based on Double Dueling Deep Q-Network (3DQN), called OTM3DQN. Based on the effective charging distance of MC, OTM3DQN cellularizes the whole network and then uses 3DQN to determine the optimal charging cell sequence from the global consideration to minimize the node dead ratio. Meanwhile, in order to improve the charging efficiency, this scheme calculates the charging amount of each cell being recharged according to the nodes’ energy demand in the cell, the network survival time, and MC’s remaining energy. Moreover, the scheme further utilizes Double DQN and Dueling DQN to reduce overestimation to speed up the training process. The main contributions of this paper are as follows:

- In contrast to existing work, this paper considers both the optimization of charging sequences and the charging amount in one-to-multiple charging. To the best of our knowledge, we present for the first time an optimization scheme that dynamically adjusts the one-to-many charging sequence and the charging amount according to the real-time network state.
- We use the 3DQN algorithm to decide the next charging cell from the candidate charging cells and to calculate the optimized amount of charging for the sensor nodes in the cell to improve the performance when avoiding node failure.
- Finally, we conducted comprehensive tests to evaluate the performance of our charging scheme under various input parameters and compared our charging scheme with existing typical schemes to determine the superiority of our charging scheme.

The paper is organized as follows: Section 2 briefly reviews the related work. In Section 3, we provide the network model and the problem formulation. Section 4 presents the detailed process of using 3DQN to optimize the charging sequences. Then, Section 5 gives the proposed charging framework and the optimization of node charge amount. In Section 6, the simulation experiment setup is described and the simulation results are discussed. Finally, Section 7 concludes the paper and presents future work.

## 2. Related Work

In recent years, there has been much research on the charging schemes for WRSN, which are classified into two main categories: offline schemes and online schemes.

### 2.1. Offline Schemes

Offline schemes usually assume that the energy consumption rate of the node is constant or varies regularly and focus on how to calculate a fixed charging sequence to achieve the minimum node dead ratio and minimum traveling distance. In order to ensure that the sensor is not dead before charging while minimizing as much as possible the number of MC, the work of [4] combined charging path optimization and depot site issues. In [5], Y. Ma et al. studied a charging strategy that maximizes the cumulative utility gain of MC while reducing the travel cost during charging and gave a constant approximation algorithm for these two problems. The authors of [6] used a multi-charger approach and provided an approximation with a proven performance ratio for the NP-hardness longest delay minimization problem. To minimize the total charging delay, C. Lin et al. combined the distance from the node to MC and the angle between the node and MC in [7] and proposed a unique energy model. In addition, they introduced a linear programming framework for determining the optimal solution and proposed a method for minimizing the complexity based on a constant approximation method. The authors of [25] proposed a bidirectional charging strategy to minimize the MC traversal path length, energy consumption, and completion time. A clustering-based approach was also proposed to reduce the total MC travel distance and reduce the energy consumed by MC charging and the total single-round completion time. The WSN was split into a few charging zones by the authors in [8]; they set the charging time to ensure that the nodes in the charging area are fully charged while minimizing the number of dead nodes. The authors of [9] cooperated to determine which sensors should be charged and when these sensors should be charged. They devised a surrogate function to characterize task collaboration. They then used the closest neighbor rule to approximate the optimization issue. Wei Z. et al. in [15] studied how to choose the optimal charging point; they used Q-learning to solve this problem.

### 2.2. Online Schemes

In the online schemes, when the remaining energy of a sensor falls below a certain level, MC will receive a charging request sent by the sensor. In [10], the authors set the charging probability for each requesting charging node; when the remaining energy of the requesting node is lower and the distance from MC is shorter, then the charging probability of the requesting charging node is higher. In [11,12], the authors developed a two-fold warning threshold charging technique to improve charging throughput. In order to reduce the charging latency, the authors of [13] proposed a gravity-searching scheme to optimize the charging order of the nodes that need to be charged. The work of [26] significantly improves charging throughput by optimizing the Fruit Fly optimization (FFO) algorithm and combining it with a threshold-based path selection algorithm to jointly optimize MC scheduling.

The authors of [16] proposed a reward function by which the reward function produces reward value to measure the performance of the charging scheme. To maximize the reward value, they used deep reinforcement learning to optimize MC scheduling and maximize the reward during the charging process. Compared with the deep reinforcement learning used by [16], Yang et al. proposed a new Actor–Critic reinforcement learning algorithm (ACRL) in [17]. In order to speed up the model training, they added the GRU layer to the Actor network, which can also schedule MC more efficiently. In [27], Banoth et al. proposed a DQN-based dynamic partial mobile charging scheduling method, where MC can interrupt the charging process of the current node, select another key node to replenish its energy, use the algorithm to predict the charging duration of the node, and collect data to train itself while performing the charging task. The authors of [28] developed a sensor energy consumption prediction model, and based on this model, the MC is efficiently scheduled, significantly improving the sensor survival rate. In [14], in order to reduce node dead ratio, the authors used fuzzy logic to establish the sequence of sensor exhaustion. The goal of [29] was to reduce the node dead ratio and increase the charging times of MC.

Several recent papers have looked at WRSN with multiple MCs [30,31]. How to coordinate and schedule MCs and cooperate with each other is the difficulty of this problem. The authors of [30] divided the network area into several regions and proposed an allocation algorithm that allowed each MC to charge several regions. In [31], in order to reduce the traveling distance by MCs, the authors proposed the idea of path merging. They made each MC responsible for a different region and assigned the same path to MC that departed for charging and MC that returned for charging.

Although a lot of works have been done on the charging scheduling of WRSN, most of the works are based on one-to-one charging technology with limited charging capacity. That leads to these works being difficult to meet the huge energy demand in large-scale WRSN. Some studies have adopted more efficient one-to-multiple charging technology to enhance charging capability. However, the schemes overlook the optimization of the charging amount that MC transfers to sensor nodes, and thus still have performance limitations. Aiming at the above problems, the paper utilizes DRL algorithms to optimize both the one-to-multiple charging sequence and the charging amount in order to further improve the charging performance in WRSN. Table 1 shows how the related work differs from our scheme.

## 3. Network Model and Problem Formulation

### 3.1. Network Structure

**Figure 2:**
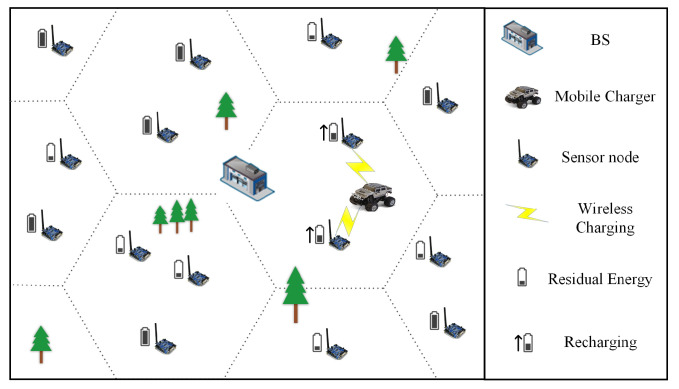
Overview of environment.

As shown in Figure 2, there are *n* sensor nodes $SN=\{sn_{1},sn_{2},\cdots ,sn_{n}\}$ and one MC in the WRSN. All sensor nodes whose locations can be accurately determined are placed on a two-dimensional map without obstacles. The location of sensor node $sn_{i}$ is $l_{i}$. We divide the two-dimensional map by a regular hexagonal cell with side length *D*; *D* is the charging range of MC. We call the hexagonal cell containing the nodes the *charging cell*. The charging cells are denoted as $Q=\{q_{1},q_{2},\cdots ,q_{m}\}$ and the center position of $q_{i}$ is denoted by $l_{q_{i}}$. The set of nodes within charging cell $q_{i}$ is $N_{i}$. The charging cell $q_{i}$ has $num_{i}$ nodes. Table 2 lists the important notations used in this paper. In the sensor network, each sensor node consumes energy when collecting data and sending data. If the node works for a long time, it may die due to insufficient energy. Due to the limited capacity of MC battery, it cannot work permanently in WRSN, and some nodes may die because they cannot obtain energy supplements in time; nevertheless, a proper charging method can minimize the node dead ratio.

### 3.2. Energy Consumption Model

The sensor node carries a battery with capacity of *E*. Each sensor node can calculate its own data acquisition frequency and data transmission rate when working and then predict its own residual energy. For each node $sn_{i}\left(1\leq i\leq n\right)$, energy is used mainly for sending and receiving data. The energy consumed for the node to send data is:

(1) $$Ts_{i}\left(t\right)=\sum_{k=1,k\neq i}^{n}m_{i,k}c_{i,k}\left(t\right)+m_{i,B}c_{i,B}\left(t\right)$$

where $c_{i,k}\left(1\leq k\leq n\right)$ and $c_{i,B}$ represent the data transfer amount an unit time from nodes $sn_{i}$ to $sn_{k}$ and B, respectively. $m_{i,k}$ is the energy required to transmit a unit of data from $sn_{i}$ to $sn_{k}$ (B). Especially, $m_{i,k}=\beta_{1}+\beta_{2}d_{i,k}^{\alpha }$; $d_{i,k}$ is the distance between $sn_{i}$ and $sn_{k}$; $\beta_{1}$ and α are hyper-parameters and fixed values. The energy consumed for a node to receive data is:

(2) $$Rp_{i}\left(t\right)=\rho \sum_{k=1,k\neq i}^{n}c_{k,i}\left(t\right)$$

the energy consumed by the received unit data is a fixed value, expressed as ρ. The final energy consumption rate of the node $sn_{i}$ is:

(3) $$r_{i}\left(t\right)=Ts_{i}\left(t\right)+Rp_{i}\left(t\right).$$

### 3.3. One-to-Multiple Charging Model

The coordinate of MC’s position is $l_{mc}$. MC moving speed is *v*, and the energy consumption is *c* per unit distance. The transmit power of MC is a fixed value, which is $U_{full}$, and MC itself carries battery energy of $E_{MC}$. MC is equipped with a wireless charging device and replenishes the nodes with wireless charging. Current wireless energy transfer technologies are classified as inductive coupling, electromagnetic radiation and magnetic resonance coupling. Since inductive coupling has limitations such as the need for close contact and accurate alignment of the charging direction, while electromagnetic radiation is less efficient, inductive coupling and electromagnetic radiation are not applicable to this paper. Therefore, the magnetic resonance coupling wireless charging technology with longer charging distance and higher charging efficiency becomes a more suitable choice.

MC replenishes energy for nodes, and when the energy of MC is insufficient, MC returns to the S to replenish its own energy. Compared with that charging process, the time for MC to replenish energy can be neglected; thus, MC cannot consume the time to replenish energy in the S, and the location of S is $l_{s}$. According to [32], we ignore the factors affecting charging efficiency and use the following one-to-multiple charging model.

When MC is in a charging cell, denote $d_{i}$ to be the distance from $sn_{i}$ to MC. All nodes in the charging cell turn on the energy receiver in preparation for charging, and MC can recharge all nodes within the charging range that are ready to be charged. Then, the received power of the $sn_{i}$ is $U_{i}=\mu \left(d_{i}\right)U_{full}$, where $\mu (d_{i})$ is the efficiency of wireless power transfer, and $\mu (d_{i})$ is the decreasing function with respect to $d_{i}$ [32], specifically:

(4) $$\mu \left(d_{i}\right)=-0.0958d_{i}^{2}-0.0377d_{i}+1.$$

According to the conclusion obtained by [33], we believe that, when MC is charging multiple nodes at the same time, the nodes do not affect each other.

### 3.4. Problem Formulation

In the problem formulation, since MC has limited energy, MC will return to the service station to replenish energy when it is low. We define the time interval between the departure of MC from the service station and its return to the service station as follows:

Definition (CR): The process from when MC leaves the service station and performs a charging task to when MC is at low energy and returns to the service station is called a charging round (CR ).

Definition (Charging latency): The time interval between the node sending a charge request message and the node starting to be charged by MC.

In this paper, the primary goal is to reduce number of dead nodes, defined as $N_{d}$. We define that a node has two states: dead and active. Thus, maximizing the proportion of active nodes while reducing the proportion of dead nodes is our primary goal. We use $dead_{i}=0$ to indicate that the $sn_{i}$ is active and $dead_{i}=1$ to indicate that the $sn_{i}$ is dead. Therefore, the goal of our scheme may be formalized as follows:

(5) $$\min N_{d}$$

subject to

(6) $$dead_{i}=\left\{\begin{array}{cc} 1, & re_{i}\leq 0\\ 0, & re_{i}>0. \end{array}\right.$$

(7) $$N_{d}=\sum_{i=1}^{n}dead_{i}$$

(8) $$E_{move}+E_{charge}\leq E$$

(9) $$U_{i}=-0.0958d_{i}^{2}-0.0377d_{i}+1.sn_{i}\in SN$$

(10) $$r_{i}=\sum_{k=1,k\neq i}^{n}m_{i,k}c_{i,k}+m_{i,B}c_{i,B}+\rho \sum_{k=1,k\neq i}^{n}c_{k,i},sn_{i}\in SN$$

where $E_{move}$ is the energy consumed by MC moving, and $E_{charge}$ is the energy consumed by MC charging nodes.

## 4. Optimized Charging Sequence

In this paper, we combine DQN, Dueling DQN, and Double DQN into the 3DQN model. In this section, we explain in detail the 3DQN network model, the construction of the learning model, and how to optimize the charging sequence by 3DQN.

### 4.1. Learning Model Construction

**Figure 3:**
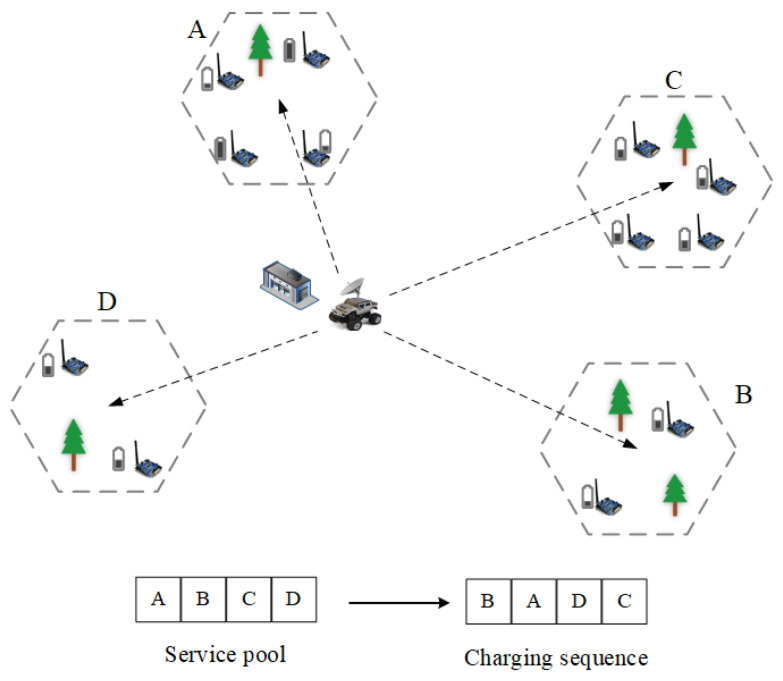
Charging sequence optimization.

MC will not perform the next charging task until it has performed one; therefore, the online one-to-multiple charging scheme is one that contains sequence of decisions. The charging process is shown in Figure 3. Suppose MC receives charging requests from A, B, C, and D, then MC scheduling is optimized by optimizing the charging decision sequence as B, A, D, and C. Since RL has a natural advantage for optimizing decision sequences, we use RL to optimize MC charging sequences.

**Figure 4:**
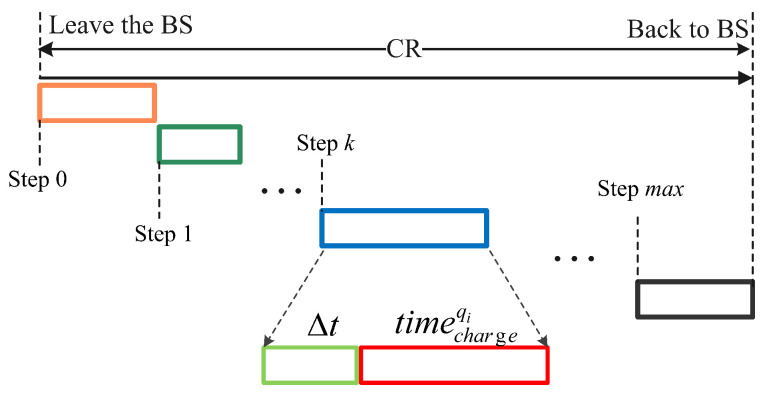
Charging sequence based on RL.

In WRSN, traditional RL is difficult to be applied to such problems because of the presence of large and high-dimensional data. However, deep learning has a powerful high-dimensional data processing capability. Therefore, RL can be combined with deep learning to form deep reinforcement learning with both high-dimensional continuous data processing capability and powerful decision-making capability, which can well solve the optimization problem of scheduling MC in WRSN. As shown in Figure 4, we have the following definition.

Definition (Step): The moment at which MC makes a charging decision based on the current state.

This paper uses state, action, and reward, and the state after the action is performed to define one-to-multiple charge scheme [34]. The states, actions, and rewards of a one-to-many charging scheme are defined below.

*State*: In online one-to-multiple charging, we take the status information of MC and nodes as the status space of WRSN, which is specifically $s=\{s_{cell},l_{mc}\}$. $l_{mc}$ is the specific position coordinate of MC. $s_{cell}$ contains node status information and charging cell location information, which is formulated as $s_{cell}=\left\{s_{q_{i}}\right\},i=1,\cdots ,m.$. $s_{q_{i}}=\left(l_{q_{i}},s_{sn_{j}}\right)$, where $s_{sn_{j}}$ is the node location and remaining energy in the $q_{i}$, specifically $s_{sn_{j}}=\left\{l_{j},re_{j}\right\},sn_{j}\in N_{i}$.

*Action*: MC makes a decision based on the current state, which is an action. In one-to-multiple charging, it is decided that the charging cell to be charged is one action, and the number of charging cells are the same as the number of action spaces.

*Reward*: The reward is obtained by MC after completing the action and is used to evaluate the action. The main objective of this paper is to optimize the scheduling of MC in order to decrease the node dead ratio in WRSN. Reducing charging latency is a secondary goal. We utilize the trip length of MC, the node dead ratio, and the request charging cell ratio in online one-to-multiple charging. The reward is defined as:

(11) $$R=e^{-(l(k)-l(k-1))}-\frac{N_{d}\left(k\right)}{n}-\frac{N_{p}\left(k\right)}{m}$$

where l(k)−l(k−1) is the distance MC moves between adjacent steps. $\frac{N_{c}\left(k\right)}{n}$ is the node dead ratio. The number of charging cells requesting charging is expressed as $N_{p}$, and *m* is the total number of charging cells.

At the initial state, MC is in the service station. When MC is working, MC will select a charging cell to charge or will go back to S due to its low energy. As shown in Figure 4, during a CR, at step *k*, the state of WRSN is $s_{k}$; thus, MC determines action $a_{k}$ and executes it. MC obtains $R_{k+1}\left(a_{k},s_{k}\right)$ and next state $s_{k+1}$ after performing the action $a_{k}$ [17]. We assume that MC chooses $q_{i}$ to charge at step *k*; in the process of MC moving and charging the charging cell $q_{i}$, the remaining nodes consume energy in the waiting process. After charging the charging cell $q_{i}$, energy updates of nodes in the charging cell $q_{i}$ are as follows:

(12) $$re_{i}\left(k+1\right)=re_{i}\left(k\right)-\sum_{t=t^{\prime }}^{t^{\prime }+\Delta t}r_{i}\left(t\right)+time_{charge}^{q_{i}}\times U_{i},sn_{i}\in N_{i}$$

The energy of the remaining nodes is updated as follows:

(13) $$re_{i}\left(k+1\right)=re_{i}\left(k\right)-\sum_{t=t^{\prime }}^{t^{\prime }+\Delta t+time_{charge}^{q_{i}}}r_{i}\left(t\right),sn_{i}\notin N_{i}$$

MC consumes energy due to movement and charging, and its energy updates are as follows:

(14) $$re_{mc}\left(k+1\right)=re_{mc}\left(k\right)-\Delta t\times c\times v-time_{charge}^{q_{i}}\times U_{full}$$

where Δt is time spent by MC to move and $time_{charge}^{q_{i}}$ is MC’s charging time in $q_{i}$. Compared to the traveling time, the movement time of MC in the charging cell can be ignored. MC will return to the service station to replenish its energy due to lack of energy. After returning to the service station to replenish its energy, MC’s energy will be updated as follows:

(15) $$re_{mc}\left(k+1\right)=E_{mc}$$

According to Formulas (12) and (13), when the node is within the charging range, its energy will increase. On the contrary, nodes not within the charging range will reduce their energy due to the waiting process. At the same time, the maximum residual energy of a node is *E* when supplementing energy, and the minimum residual energy of a node is 0 when consuming energy.

We applied some criteria for training and creating suitable solutions, namely:

1. When the remaining energy of MC is sufficient, MC can charge any of the charging cells.
2. MC does not consume energy when not moving and not charging.
3. MC does not accept charging requests during the charging process.
4. MC cannot be interrupted during the charging process.
5. If the residual energy of MC cannot support the charging task, it must return to the S to replenish the energy.

### 4.2. Deep Q-Networks

Q-learning is the classical method for charging sequential decision problems [15]. Under the following policy π, the expected value of the total return obtained by choosing a series of actions $\left\{a_{0},a_{1},a_{2},\cdots \right\}$ is called the Q-value, and then the sequential decision problem can be solved by learning bootstrap estimates based on the Q-value. An action *a* in state *s* follows the ground-truth value of policy π as

(16) $$Q_{\pi }\left(s,a\right)=E\left[\sum_{i=1}\gamma^{i-1}r_{i}|s_{0}=s,a_{0}=a,\pi \right]$$

where γ∈[0,1] represents the discount factor. The discount factor’s function is to provide incentives received at various points in the future with various weights and values. The best value is $Q^{*}\left(s,a\right)=max_{\pi }Q_{\pi }\left(s,a\right)$. Since the Q-table can only calculate Q-values for a limited state space, it is difficult to apply in the constantly varying WRSN environment; thus, deep neural networks are used as a nonlinear function to approximate Q-values, i.e., deep neural networks are combined with classical Q-learning to form a deep Q-network (DQN).

The DQN improves training efficiency and reduces fluctuations by means of experience replay buffer B and dual networks [35]. The neural network used to estimate the Q-value function is the main network with parameters of $\theta^{+}$, and the neural network used to output iterative learning target values is the target network with parameters of $\theta^{-}$. We randomly take a certain number of *b* groups of samples from the B for gradient descent calculation to train the main network and eliminate the correlation between the sample data to improve the learning efficiency. The target value of iterative learning is expressed as:

(17) $$y_{i}^{DQN}=R_{i+1}+\gamma \underset{a}{max}Q\left(s_{i+1},a;\theta_{i}^{-}\right).$$

The main network parameter update formula is as follows:

(18) $$\theta_{i+1}^{+}=\theta_{i}^{+}+\eta \left[y_{i}^{DQN}-Q\left(s_{i},a_{i};\theta_{i}^{+}\right)\right]\nabla_{\theta_{i}^{+}}Q\left(s_{i},a_{i};\theta_{i}^{+}\right)$$

where η∈[0,1) is the learning rate; the main network updates the parameters to the target network every *n* steps.

### 4.3. Double Dueling Deep Q-Networks

To pick and evaluate the greedy action, the maximized operators in Formula (17) employ identical Q values. Since action selection and evaluation use the same network, it is more likely that an overestimation of the true action value is generated because of bootstrapping. The double deep Q-network (DDQN) [36] has two DQN networks; the main DQN network outputs the actions and the target DQN network computes the target values for iterative learning to avoid bootstrapping and reduce overestimation. Therefore, the DDQN updates the objective as:

(19) $$y_{i}^{DDQN}=R_{i+1}+\gamma Q\left(s_{i+1},arg\underset{a}{max}Q\left(s_{i+1},a;\theta_{i}^{+}\right);\theta_{i}^{-}\right).$$

It can be seen from Formula (19) that we use the main network to select the action and the target network to participate in the calculation of the updated target value. The main network parameters are likewise updated to the target network every *n* steps.

**Figure 5:**
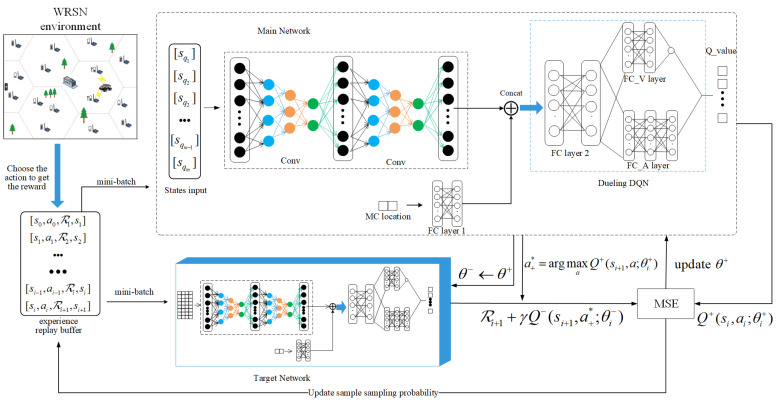
Network model structure of the 3DQN.

In order to further improve its steady-state control, we combine DDQN and Dueling DQN [37] to produce the Double Dueling Deep Q-Network (3DQN) to attain better outcomes. We further stabilize the training process with 3DQN, which contains two different estimators: V(s) is used to estimate the state value and A(s,a) is the action advantage function. Finally, the output values of the two functions are combined as follows:

(20) $$Q\left(s,a\right)=V\left(s\right)+(A\left(s,a\right)-\frac{1}{\left|A\right|}\sum_{a^{\prime }}A\left(s,a^{\prime }\right))$$

where V(s) is a scalar and A(s,a) is a |A|-dimensional vector, the V(s) and A(s,a) are output by two independent streams, and the last hidden layer of the original DQN is divided into these two independent streams. The suggested 3DQN’s design is shown in Figure 5. The main network is in the upper part of Figure 5, and the target network is in the lower part of Figure 5. Two DQNs have Dueling structures and are jointly trained to represent two Q-valued functions, but they are structurally independent.

### 4.4. Training with 3DQN

As shown in Figure 5, we combine the $l_{q_{i}}$ and the $s_{sn_{j}},sn_{j}\in N_{i}$ into $s_{q_{i}},i=1,\cdots ,m$, which is the input of the convolution layer. After passing through the fully connected layer, MC position information is combined with the convolution layer’s output as the next layer’s input.

In 3DQN, the main network is represented by $Q^{+}\left(\cdot ;\theta^{+}\right)$, and the target network is represented by $Q^{-}\left(\cdot ;\theta^{-}\right)$. At each step, the $a_{k}$ generated by the $\varepsilon_{k}$-greedy policy is executed, and the value of $\varepsilon_{k}$ decreases monotonically with the training process, with a minimum value of 0.01. Then, through the reward function, we obtain the reward value $R_{k+1}$, and obtain the next state $s_{k+1}$. We store $s_{k},a_{k},R_{k+1},s_{k+1}$ as a tuple $\left(s_{k},a_{k},R_{k+1},s_{k+1}\right)$ into B.

In the process of training, a certain number of samples are randomly taken from the B for gradient descent calculation each time, which is less efficient in learning the differences of effects produced by different actions in the samples. Therefore, a weighted sample pool (Prioritized experience replay) [38] is introduced to set different sampling weights for different samples. The worse the performance of an action in a sample, i.e., the greater the deviation relative to the estimate, the greater the probability that it will be selected by the action to improve learning efficiency. In this paper, we use a stepwise approach to calculate the sampling weights of the samples. The estimation error of sample *i* is as follows:

(21) $$\delta_{i}=\left|Q{\left(s,a;\theta^{+}\right)}_{i}-Q{\left(s,a;\theta^{-}\right)}_{i}\right|.$$

The sampling weight of the sample set is the inverse of the estimation error of the sample, and then the sampling probability of sample *i* can be obtained as:

(22) $$P_{i}=\frac{\frac{1}{\delta_{i}^{\tau }}}{\sum_{k\in M}\frac{1}{\delta_{k}^{\tau }}}$$

where τ indicates the magnitude of the action of the sampling weights. When τ=0, the weighted sampling becomes ordinary random sampling. The OTM3DQN training is described in detail in Algorithm 1.   
**Algorithm 1:** OTM3DQN
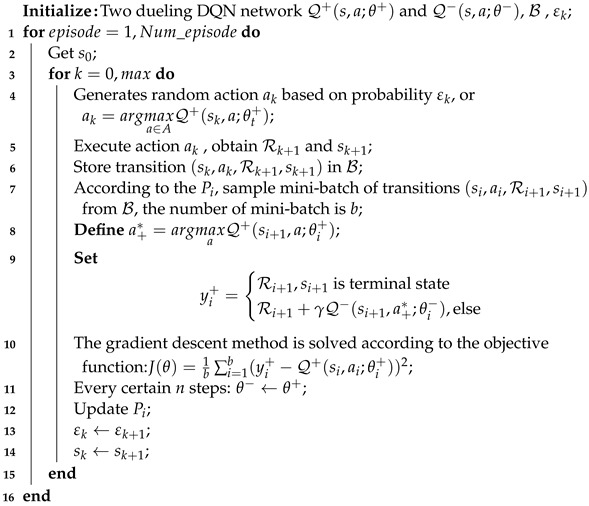


## 5. Charging Scheme Details and Procedures

This section describes the specific steps of the charging scheme in this paper, which includes how to calculate the charging request threshold and the node charging amount, which are aimed at reducing the sensor node dead ratio. For the former, we calculate the node charging threshold by computing the node’s minimum tolerated charging time and determining the conditions under which charging cells send charging requests based on the node charging threshold. In particular, the charging requests for the charging cells are sent by the aggregation nodes. For the node charging amount, we calculate it from three perspectives: individual node demand, average residual time of all nodes in the whole WRSN, and MC’s residual energy, which changes dynamically according to the network state. Then the center of gravity method is used to adjust the charging location based on the node charging amount. In addition, since MC energy is limited, MC will determine whether its energy is sufficient before performing the charging task. Section 5.2 describes the charging request threshold. Section 5.3 and Section 5.4 calculate the node charging amount and MC charging location. Section 5.5 determines whether MC performs the next charging task.

### 5.1. The Charging Scheme Overview

Our charging scheme is described as follows: we use MC effective charging distance as the side length of the regular hexagonal division network; the hexagon with internal nodes is named as the *charging cell* and expressed as $q_{1},q_{2},\cdots ,q_{m}$. MC maintains the 3DQN algorithm and a service pool C, and when a charging cell reaches the charging request condition, it will send a message to MC with the request to charge, then MC receives this message, updates the service pool, and then performs the following operations:

- MC obtains the optimal or nearly optimal charging cell $q_{i}$ by using 3DQN algorithm;
- MC determines whether to go to charging cell $q_{i}$ according to Section 5.5;
- After MC reaches the center of the charging cell $q_{i}$, the charging amount of nodes within the charging cell is derived according to Section 5.3, and the charging position is derived according to Section 5.4;
- MC decides whether to charge the nodes in charging cell $q_{i}$ according to Section 5.5;

As the proposed scheme is non-preemptive, MC performs a new charging task only after the current charging task is completed. After performing the charging task, MC obtains the current WRSN state as an input to the 3DQN algorithm and derives the next optimal charging cell.

### 5.2. Charging Request Threshold

The charging request threshold (defined as $E^{thred}$) determines when a cell sends a charging request. The node residual energy reaches threshold when the node residual energy falls below a certain level, so the threshold $E_{i}^{thred}$ for node $v_{i}$ is

(23) $$E_{i}^{thred}=\theta E$$

where θ is the threshold factor. If whole charging cell is considered, a simple analysis from Formula (23) shows that the threshold for sending a charging request from the charging cell $q_{i}$ should be a simple sum of the thresholds of each node:

(24) $$E_{q_{i}}^{thred}=\sum_{sn_{i}\in N_{i}}E_{i}^{thred}\;$$

This paper takes into account that, when more than half of the number of sensor nodes within the charging cell residual energy drop below the threshold, or the value of the residual energy within the charging cell is below the $E_{q_{i}}^{thred}$, then this charging cell sends a charging request. Therefore, the service pool of MC is expressed as:

(25) $$C=\{q_{i}|\forall sn_{i}\in N_{i},\sum_{sn_{i}\in N_{i}}re_{i}\leq E_{q_{i}}^{thred}\}.$$

### 5.3. Charging Amount of Nodes

In previous studies, both the full and partial charging schemes of sensor nodes have significant limitations which cannot meet the dynamic WRSN environment. The charging amount of node $E_{i}^{c}$ is discussed from three aspects below.

(1) Analysis from individual sensor node

Since lowering the node dead ratio as much as feasible is the aim of this paper, the energy replenished to each sensor node should be as much as possible to ensure that it does not die before the next charging service; thus, the value of $E_{i}^{c}$ for $sn_{i}$ is taken as follows:

(26) $$E_{i}^{c}=E-re_{i}.$$

The charging amount $E_{q_{i}}^{c}$ of the $q_{i}$ where $sn_{i}$ is located is:

(27) $$E_{q_{i}}^{c}=\sum_{sn_{i}\in N_{i}}E_{i}^{c}.$$

(2) Analysis from all charging cells

When charging the charging cell, the energy variation of the sensor nodes in the other charging cells should be considered by MC and it should not lose sight of them. After MC finishes this charging task, MC still needs to go to $q_{1},q_{2},\cdots$. We use $d_{q_{i}q_{j}}$ to represent the distance between $q_{i}$ and $q_{j}$ centers; thus, the distance matrix between the centers of charging cells in WRSN is:

(28) $$\left[\begin{array}{cccc} d_{q_{1}q_{1}} & d_{q_{1}q_{2}} & \cdots & d_{q_{1}q_{m}}\\ d_{q_{2}q_{1}} & d_{q_{2}q_{2}} & \cdots & d_{q_{2}q_{m}}\\ \vdots & \vdots & \ddots & \vdots \\ d_{q_{m}q_{1}} & d_{q_{m}q_{2}} & \cdots & d_{q_{m}q_{m}} \end{array}\right]$$

The matrix column summation is:

(29) $$\left[\begin{array}{c} \sum_{i=1}^{m}d_{q_{1}q_{i}}\\ \sum_{i=1}^{m}d_{q_{2}q_{i}}\\ \vdots \\ \sum_{i=1}^{m}d_{q_{m}q_{i}} \end{array}\right].$$

Then, Matrix row summation is $\sum_{i=1}^{m}\sum_{j=1}^{m}d_{q_{i}q_{j}}$. Thus, the average distance between the center positions of any two charging cells is:

(30) $$d=\frac{1}{m^{2}}\sum_{i=1}^{m}\sum_{j=1}^{m}d_{q_{i}q_{j}}$$

We replace the average length of the movement in MC charging task with *d*. Considering only the latency of MC movement, the average waiting time of sensor nodes in the network waiting for MC charging is:

(31) $$time_{w}=\frac{\left[\frac{d}{v}+\frac{2d}{v}+\cdots +\frac{md}{v}\right]}{m}=\frac{\left(m+1\right)\sum_{i=1}^{m}\sum_{j=1}^{m}d_{q_{i}q_{j}}}{2vm^{2}}.$$

The overall residual energy of the $q_{i}$ is expressed as $re_{q_{i}}=\sum_{sn_{i}\in N_{i}}re_{i}$. Similarly, the received power of the $q_{i}$ is $U_{q_{i}}=\sum_{sn_{i}\in N_{i}}U_{i}=U_{full}\sum_{sn_{i}\in N_{i}}\mu \left(distance\left(l_{i},l_{q_{i}}\right)\right)$. The average energy surplus of charging cells in the WRSN is:

(32) $$RE=\frac{\sum_{i=1}^{m}re_{q_{i}}}{m}.$$

We use the total energy consumption rate of the internal nodes of the charging cell to represent the energy consumption rate of the charging cell. Therefore, the energy consumption of $q_{i}$ is $r_{q_{i}}=\sum_{sn_{i}\in N_{i}}r_{i}$, and the energy consumption rate of charging cells in the WRSN is:

(33) $$R=\frac{\sum_{i=1}^{m}r_{q_{i}}}{m}.$$

Then the average network survival time is $time_{l}=RE/R$. So that not too many sensor nodes die in the network, there is the following equation:

(34) $$\frac{E_{q_{i}}^{c}}{U_{q_{i}}}+time_{w}=time_{l}.$$

The equation can be rectified to obtain:

(35) $$E_{q_{i}}^{c}=\left(time_{l}-time_{w}\right)\times U_{q_{i}}.$$

After obtaining the energy required for the charging cell, the energy required for each node within the charging cell can be calculated. In this paper, according to the principle that, the longer the survival time, the less energy is allocated, and the survival time of $sn_{i}$ is $time_{i}^{l}=re_{i}/r_{i}$, therefore, the energy that should be allocated to $sn_{i}$ in $q_{i}$ is:

(36) $$E_{i}^{c}=\frac{e^{-time_{i}^{l}}}{\sum_{sn_{i}\in N_{i}}e^{-time_{i}^{l}}}\times E_{q_{i}}^{c}.$$

(3) Analysis from MC residual energy

To minimize the node dead ratio and simultaneously increase MC’s energy efficiency, the service time of MC to perform a charging task must be manageable. The average charging efficiency of the charging cells in the network is:

(37) $$U=\frac{\sum_{i=1}^{m}U_{q_{i}}}{m}.$$

Assume that the number of charging cells in the service pool requesting charging is *x*. Therefore, we have the following equation:

(38) $$\begin{array}{cc} re_{mc}= & \frac{E_{q_{i}}^{c}}{U}\times U_{full}\times x+\left(d+2d+\cdots +xd\right)\times c\\ \; & =\frac{E_{q_{i}}^{c}}{U}\times U_{full}\times x+dc\sum_{i=1}^{x}i \end{array}$$

where $re_{mc}$ is the residual energy of MC, the $E_{q_{i}}^{c}$ of $q_{i}$ is obtained as:

(39) $$\begin{array}{cc} E_{q_{i}}^{c} & =\frac{re_{mc}-dc\sum_{i=1}^{x}i}{U_{full}x}\times U\\ \; & =[\frac{re_{mc}-\left[\frac{x(x+1)}{2m^{2}}\sum_{i=1}^{m}\sum_{j=1}^{m}d_{q_{i}q_{j}}\right]c}{U_{full}\times x}]\times U. \end{array}$$

According to the above analysis, the value of $E_{q_{i}}^{c}$ should be the median of the values taken by the three formulas of (27), (35), and (39). If the value of $E_{q_{i}}^{c}$ is greater than the value found in Formula (27), then $E_{i}^{c}=E-re_{i}$. Otherwise, the value of $E_{i}^{c}$ is obtained according to Formula (36), and the energy is allocated according to the principle of energy consumption rate of sensor nodes. Algorithm 2 presents the algorithm’s pseudo-code.

### 5.4. Mc Charging Location Determination

Through the analysis in Section 5.3, MC obtains the charging amount of node. Then, MC determines the appropriate charging location to replenish nodes in the charging cell with the energy they need. The coordinates of $sn_{i}$ in the $q_{i}$ in 2D are $\left(x_{i},y_{i}\right)$, and the required energy $E_{i}^{c}$ is the weight; the center of gravity of the object in the solution plane are:

(40) $$\left\{\begin{array}{c} x_{0}\times \sum_{sn_{i}\in N_{i}}E_{i}^{c}=\sum_{sn_{i}\in N_{i}}\left(x_{i}\times E_{i}^{c}\right)\\ y_{0}\times \sum_{sn_{i}\in N_{i}}E_{i}^{c}=\sum_{sn_{i}\in N_{i}}\left(y_{i}\times E_{i}^{c}\right). \end{array}\right.$$

Therefore, the adjusted charging position $l_{q_{i}}^{c}$ of MC after reaching the $q_{i}$ to be charged is:

(41) $$\left\{\begin{array}{c} x_{0}=\frac{\sum_{sn_{i}\in N_{i}}\left(x_{i}\times E_{i}^{c}\right)}{\sum_{sn_{i}\in N_{i}}E_{i}^{c}}\\ y_{0}=\frac{\sum_{sn_{i}\in N_{i}}\left(y_{i}\times E_{i}^{c}\right)}{\sum_{sn_{i}\in N_{i}}E_{i}^{c}}. \end{array}\right.$$

**Algorithm 2:** Charging amount of nodes determination
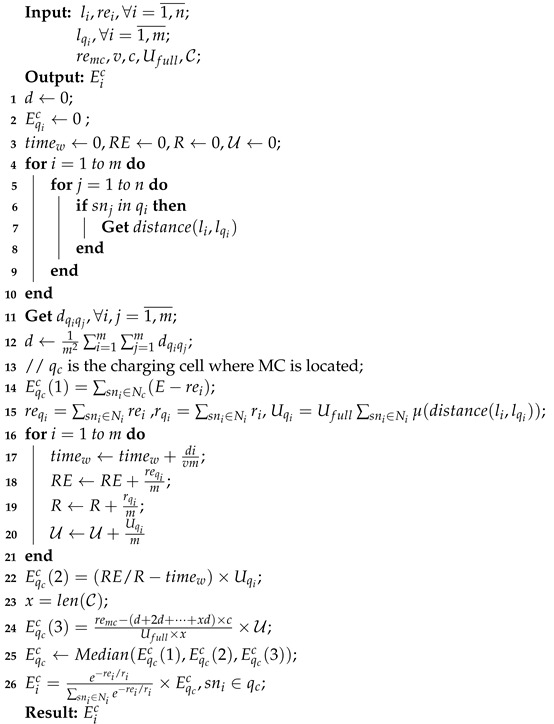


### 5.5. Analyze Whether MC Returns to the S

After selecting the next charging cell to be recharged, MC will check whether it has enough energy before recharging. MC can check whether its energy is enough in two steps. First, MC can judge whether its energy is enough to go to the charging cell $q_{i}$ center $l_{q_{i}}$ and return to the S. The energy for MC to move from the current position to the center of $q_{i}$ is

(42) $$E_{l_{mc}\to l_{q_{i}}}=distance\left(l_{mc}l_{q_{i}}\right)c.$$

The energy returned to the S from the charging cell $q_{i}$ center is

(43) $$E_{l_{q_{i}}\to l_{s}}=distance\left(l_{q_{i}},l_{s}\right)c.$$

If $re_{mc}\leq E_{l_{mc}\to l_{q_{i}}}+E_{l_{q_{i}}\to l_{s}}$, MC returns to S and this CR ends. Otherwise, MC heads to the charging cell center.

If MC has enough energy to return to S, it should also be determined whether MC has enough energy to perform the charging task. The energy of MC moving from the charging cell $q_{i}$ center $l_{q_{i}}$ to the charging position $l_{q_{i}}^{c}$ is $E_{l_{q_{i}}\to l_{q_{i}}^{c}}=distance\left(l_{q_{i}},l_{q_{i}}^{c}\right)c$. The charging time $time_{q_{i}}^{c}$ of MC in the $q_{i}$ is

(44) $$time_{q_{i}}^{c}=max\left[\frac{E_{i}^{c}}{\mu \left(distance\left(l_{mc},l_{i}\right)\right)\times U_{full}}\right]sn_{i}\in N_{i}.$$

The $E_{q_{i}}=time_{q_{i}}^{c}U_{full}$ energy consumed by MC replenishes energy for sensor nodes in $q_{i}$. From the charging position of the $q_{i}$, the energy returned by MC to the S is $E_{l_{q_{i}}^{c}\to l_{s}}=distance\left(l_{q_{i}}^{c},l_{s}\right)c$. Therefore, if $re_{mc}\leq E_{q_{i}}+E_{l_{q_{i}}^{c}\to l_{s}}+E_{l_{q_{i}}\to l_{q_{i}}^{c}}$, MC returns to S and this CR ends. Otherwise, the nodes in the charging cell are charged by MC. The specifics are explained in Algorithm 3.
**Algorithm 3:** Whether MC returns S
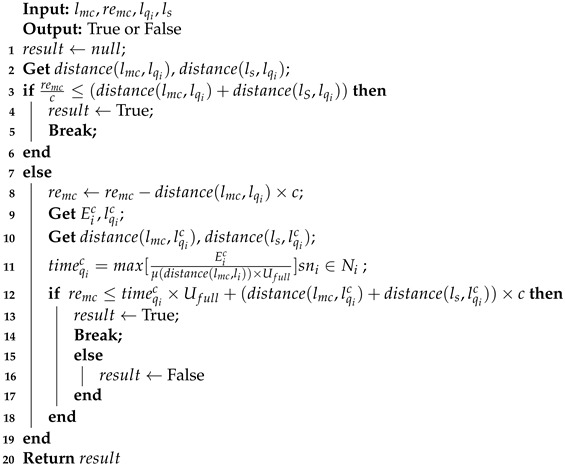


## 6. Experiment

In this section, we analyze our proposed scheme through experiments. We provide further experimental information, along with a comparison of several approaches.

### 6.1. Experimental Settings

We take into account three networks of varying sizes, with 50, 100, and 200 sensor nodes. *A* 3.7 V/450 mAh alkaline rechargeable battery powers the node. Therefore, maximum energy capacity of node is *E* = 3.7 V × 0.45 A × 3600 s = 6 kJ. In that initial state of the network environment, the node positions are uniformly and randomly drawn in the square area with a side length of 1000 m, and the BS is located at (500,500). The energy of the node is randomly distributed between 500 and 6000 J. The capacity of MC is $E_{mc}$ = 25 × 3.7 V × 3 A × 3600 s = 1000 kJ since it has 25 Li-ion batteries, each of which has a voltage and power of 3.7 V and 3000 mAh, respectively. The speed of MC is 5 m/s, the transmit power of MC is 20 w, and the energy consumption of MC is 50 J/m. We use the Python simulator (Python v3.6.8) and Pytorch 1.7.0 to build the simulation environment

In the training process, we use a mini-batch size of 32 as the input, 1D convolution layer as the input layer of the neural network, and the dimension of the input layer is the same as that of the state *s*. We combine the MC position information processed by the fully connected layer with the state-of-nodes information processed by the convolutional layer as the input of the following fully connected layer.

The main network has four hidden layers: two convolutions, one fully connected layer, and two parallel fully connected layers outputting *A* and *V*, respectively. The number of output channels of *A* is the same as the action space, and *V* has only single output channel. Finally, these two values are calculated by Formula (20) to obtain the Q. All networks use the ReLU activation. We used the Adam optimizer to train our model, and the 1 ×$10^{-6}$ learning rate was applied to the training of all scale networks. The target network structure is the same as the main network structure. The OTM3DQN is trained for 1200 CRs on 100 nodes, 150 nodes, and 200 nodes, respectively. The experimental parameters are shown in Table 3.

### 6.2. Result of Comparison with Others

We carry out comparison experiments to show the performance of our proposed scheme, OTM3DQN. The charging schemes we compare include the Greedy scheme and the FCFS scheme. We apply two deep reinforcement learning algorithms RMP-RL [16] and ACRL [17] in this paper, they schedule MC to move to center of charging cell and apply the fully charging method. The FCFS scheme and the Greedy scheme are introduced as follows.

(1) *FCFS scheme*: In the FCFS scheme, when the charging cell meets the conditions for entering MC service pool, the charging cell sends a charging request. Then, MC inserts the charging cell identification that sent the charging request into C after receiving the request. Charging cells are selected on a first-come, first-served basis. MC selects a charging cell from C and goes to the center of the charging cell to fully charge all nodes in the charging cell. After charging is complete, MC removes the charging cell identification from C and continues to receive charging requests.

(2) *Greedy scheme*: In the Greedy scheme, when the charging cell meets the conditions for entering the MC service pool, the charging cell sends a charging request. Then, MC inserts the charging cell identification that sent the charging request into C after receiving the request. At each step, with the Greedy scheme min $distance\left(l_{q_{j}},l_{q_{i}}\right)+re_{q_{j}}$, MC selects a charging cell from C and goes to the center of the charging cell to fully charge all nodes in the charging cell. After charging is complete, MC removes the charging cell identification from C and continues to receive charging requests.

**Figure 6:**
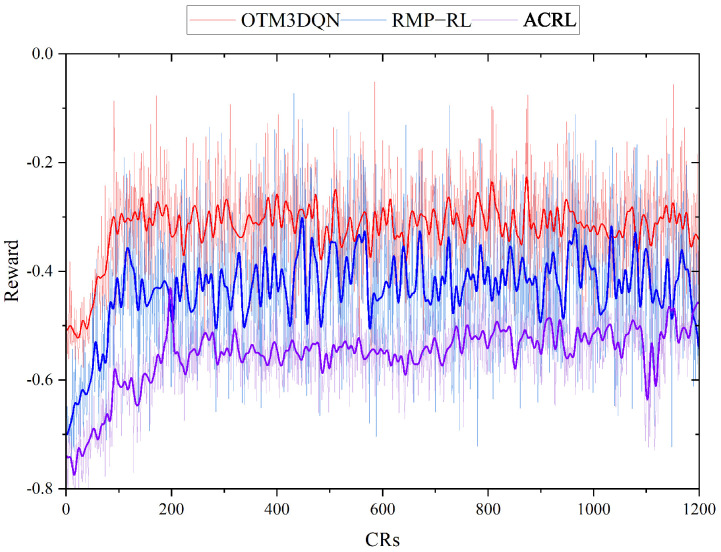
Training process average reward value for OTM3DQN, RMP-RL, and ACRL.

We first compare the performance of two deep reinforcement learning algorithms RMP-RL and ACRL in training; these two schemes were studied for 1200 CRs. Figure 6 shows the change in reward values during training for OTM3DQN, RMP-RL, and ACRL at the scale of 100 nodes, MC speed of 5 m/s, and transmit power of 20 w. As shown in Figure 6, the performance of OTM3DQN is the highest and most stable. OTM3DQN has a higher reward value because MC supplements a more reasonable energy value for the node when charging the node, thus reducing charging time which can replenish energy for other nodes in time and reduce node dead ratio. Meanwhile, OTM3DQN is more stable and faster than RMP-RL because OTM3DQN uses Double DQN to alleviate the overestimation caused by *bootstrap* operation during training, and then uses Dueling DQN to improve the stability of training, which makes OTM3DQN obtain a faster convergence rate and better stability.

ACRL has the best stability during training, but the reward value is lower than RMP-RL because RMP-RL selects actions based on value. In contrast, ACRL selects actions based on probability during training. In the process of training, when performing an action yields a higher reward, ACRL will increase the probability of the action choice; this will give ACRL a greater probability of selecting some of the better-performing actions. Therefore, the ACRL training has good stability, but it can also lead to the appearance of fear forward and fear backward when facing unknown strategies. With unknown strategies, this makes it possible that there are better policies that are not being executed, which is the reason for the lower ACRL reward value.

### 6.3. Impact of MC Transmitting Power

**Figure 7:**
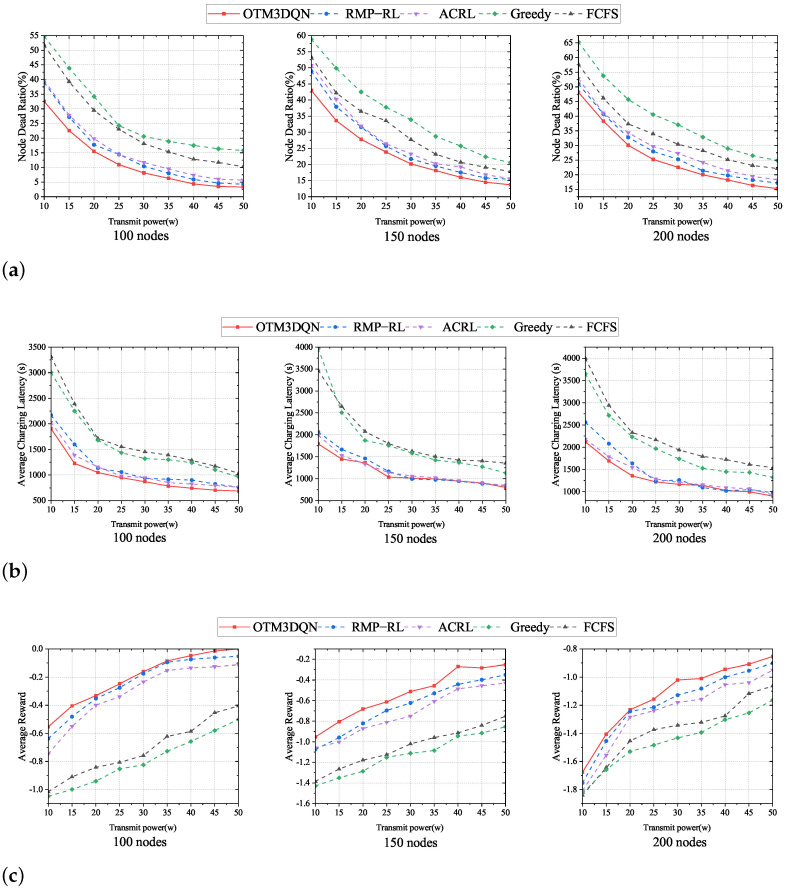
Impact of MC transmitting power. (**a**) Node dead ratio under different MC transmitting power. (**b**) Average charging latency under different MC transmitting power. (**c**) Average reward under different MC transmitting power.

We mainly talk about how MC transmitting power $U_{full}$ affects charging effectiveness. The movement speed of MC is fixed at 5 m/s. The value of $U_{full}$ uniformly increases from 10 w to 50 w. We count the number of dead nodes at the completion of each charging task of the MC and calculate the node dead ratio when MC returns to S. In Figure 7, we show the comparison results with OTM3DQN, RMP-RL, ACRL, Greedy, and FCFS in networks with different numbers of sensor nodes.

As depicted in Figure 7a,b, in 100, 150, and 200 nodes, the three different network sizes, we observe that node dead ratio and average charging latency are relatively high at the beginning at lower $U_{full}$, and node dead ratio and average charging latency show decreasing trend as $U_{full}$ increases. The reason is that, when $U_{full}$ is too small, resulting in longer charging time, the residual energy of subsequent nodes rapidly decreases, leading to more nodes with insufficient energy, a sharp increase in charging requests, a sharp increase in node dead ratio, and a rapid increase in the average charging latency. With the gradual increase of $U_{full}$, MC can replenish energy for nodes faster, with more energy remaining in subsequent nodes and shorter waiting time for charging, leading to a lower node dead ratio and average charging latency.

It can be seen from Figure 7a,b that OTM3DQN has the lowest node dead ratio and the lowest average charging latency among the five charging schemes. This is because OTM3DQN uses the 3DQN algorithm to select the optimal charging cell from the global perspective, rather than just calculating the next charging cell from the data in the C, which takes into account the nodes that are likely to fall into energy deficit, thus reducing the node dead ratio. At the same time, OTM3DQN analyzes the three aspects to derive a reasonable node charging amount, thus making MC not spend too much time charging, allowing subsequent nodes to wait less time, and also then renewing nodes to have more energy to support until the next charge, which results in less node dead ratio and average charging latency.

Although the reward value function is used to guide the deep reinforcement learning training process, Greedy and FCFS can still derive data, such as the number of dead nodes, which can be substituted into the reward function to derive the reward value. To unify the reward values, we use *n* for 100 and *m* for 32. In Figure 7c, comparing the three network scales, we observed that the average reward for all five charging schemes tends to increase as $U_{full}$. This is because, when $U_{full}$ increases, nodes can replenish their energy faster. The remaining nodes will have a shorter waiting time, resulting in fewer dead nodes and requesting cells and a rise in the average reward value.

We find that there is no optimal value of transmitting power, but after reaching a specific value, the tendency of various indexes to improve with increasing transmitting power becomes slower. For a network size of 100 nodes using the OTM3DQN charging scheme, this value is about 35 w, which varies for different network sizes and charging schemes, but is about 25 w to 40 w, and this range should continue to increase as the network size continues to expand. We can also see that all three charging schemes using deep reinforcement learning outperform the traditional charging schemes, with the OTM3DQN charging scheme performing the best. It can also be seen in Figure 7c that the traditional charging schemes have substantial limitations due to the overemphasis on one aspect, such as the Greedy, because the Greedy charging scheme averages the traveling distance weight and the residual energy weight of the nodes and focuses only on the charging cells in the C, making the Greedy charging scheme short-sighted.

### 6.4. Impact of MC Speed

**Figure 8:**
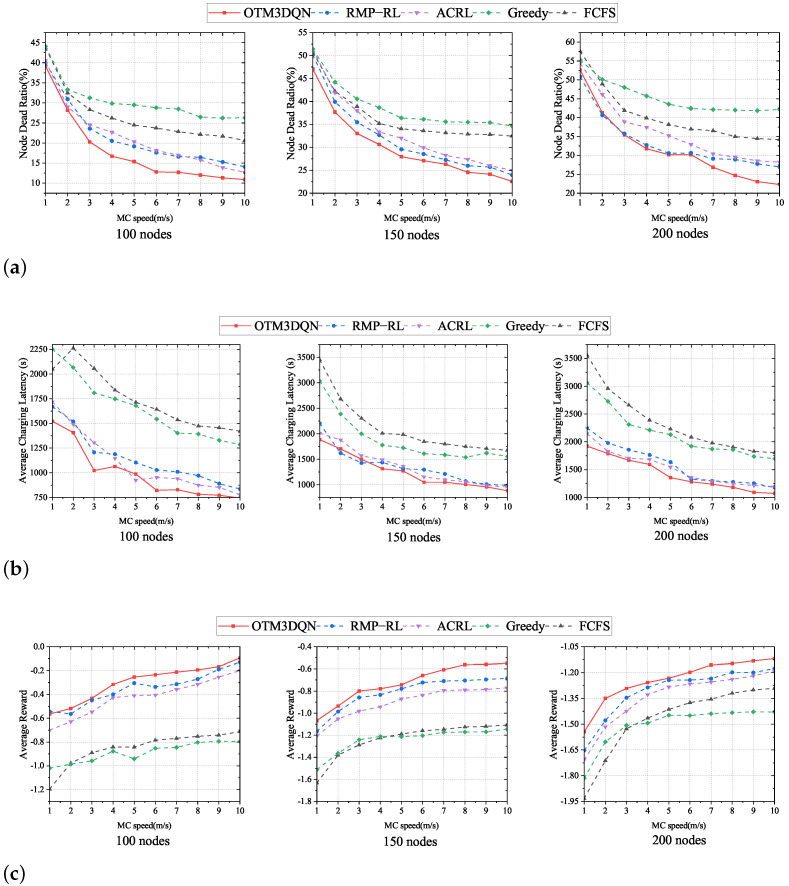
Impact of MC speed. (**a**) Node dead ratio under different MC speed. (**b**) Average charging latency under different MC speed. (**c**) Average reward under different MC speed.

We analyze the performance of the three metrics for five charging schemes with a range of MC speed as a sequence of arithmetic progression from 1 m/s to 10 m/s, and we fix MC transmitting power $U_{full}$ is 20 w. From Figure 8, we can observe that the node dead ratio and average charging latency decrease with the increase of MC movement speed. The average reward also increases with the increase of MC movement speed. The OTM3DQN continues to be the best performer among the five charging schemes, again because OTM3DQN uses the 3DQN algorithm and analyzes the node charging amount, making the selection of charging cells and charging amounts more reasonable, and thus reducing node dead ratio and average charging latency.

It can be seen from Figure 8 that the performance of FCFS and Greedy charging schemes almost do not improve with the increase of MC speed after reaching a specific value, which is due to the short-sightedness caused by the limitations of FCFS and Greedy. Since FCFS only focuses on the residual energy, and Greedy, although it considers both the traveling distance and the residual energy, cannot dynamically adjust the weights between the traveling distance and the residual energy, MC only moves between a few charging cells under these two schemes, thus making FCFS and Greedy fall into local optimal solutions.

Meanwhile, in Figure 8c, it can be observed that, when MC speed is low, the enhancement of the performance of the charging schemes by increasing the speed is pronounced. However, as MC speed increases, the enhancement of charging schemes’ performance by increasing the speed is too limited. It can be concluded that, when MC speed reaches a specific range, it is difficult to improve the charging efficiency by increasing MC speed.

### 6.5. Charging Cost and Energy Efficiency

Table 4 shows the average travel length and energy efficiency obtained by different schemes for 100, 150, and 200 nodes, MC speed of 5 m/s, and transmit power of 20 w. The average travel length is called average cost. *Energy efficiency* is the ratio of the replenishment energy of the sensor node to the maximum available energy of MC in CR. We observe that the OTM3DQN charging scheme outperforms the remaining four charging schemes in terms of energy efficiency and average cost. In contrast, the Greedy charging scheme performs just below the OTM3DQN charging scheme in terms of energy efficiency over 100 and 150 nodes. This is because the Greedy charging scheme averages the traveling distance weight and the residual energy weight of the nodes and focuses only on the charging cells in the C, making the Greedy charging scheme short-sighted. In other words, the OTM3DQN charging scheme is more long-term by dynamically adjusting the traveling distance weight and node residual energy weight from a global perspective. The FCFS charging scheme performs the worst since the FCFS charging scheme only focuses on the residual energy of the nodes. As a result, MC charges only a few charging cells in WRSN, which consumes much energy during the movement, leading to higher average cost and lower energy efficiency. We also observe that the Greedy and FCFS charging schemes perform worse as the network size increases, while the OTM3DQN charging scheme performs better, which proves the superiority and scalability of our charging scheme.

## 7. Conclusions and Future Work

In this paper, we have proposed a novel one-to-multiple charging scheduling optimization scheme based on DRL, called OTM3DQN, for WRSN. Different from the existing one-to-multiple works that only optimize the charging sequence, OTM3DQN explicitly analyzes the charging amount that MC transfers to the sensor node(s) in each charging task and utilizes Double Dueling DQN (3DQN) to optimize the scheduling of charging sequences of MC in order to improve the overall charging performance. The proposed scheme further utilizes Double DQN to reduce overestimation caused by bootstrapping to lower the error and thus achieve better performance. In addition, OTM3DQN makes use of the Dueling DQN structure to improve the stability of training and uses the prioritized experience replay to improve the training efficiency. Extensive simulation experiments have been conducted to evaluate the performance of the proposed scheme. The results show that, in large-scale WRSN, OTM3DQN has better performance in reducing node dead ratio and charging latency compared with several existing typical works.

In the future, we will work on complex WRSN with multiple MCs to achieve lower node mortality for a larger network. Our goal also includes applying the algorithm to real-world environments in the future. 

## Figures and Tables

**Table 1:** Comparison of our charging scheme with existing schemes.

Category	Charging Scheme	Charging Amount	Optimization Objective	Optimization Scheme	Paper
offline	One-to-One	fully charging	Charging path	Traditional heuristic method	[5,9,19]
			Charging path, depot location	Traditional heuristic method	[4]
			Charging path	RL	[15]
Online			Charging sequence	Traditional heuristic method	[10,12,13]
			Charging sequence	Classical optimization method	[26]
			Charging sequence	DRL	[27]
	One-to-multiple	fixed ratio charging	Charging sequence	Traditional heuristic method	[14,29]
		dynamic charging amount	Charging sequence, Charging amount	DRL	Our charging scheme

**Table 2:** List of important notations.

Notation	Definition
*n*	Number of sensors
*m*	Number of charging cells
$l_{i}$	Location of sensor node *i*
$l_{q_{i}}$	Central location of the charging cell $q_{i}$
$N_{i}$	Set of sensor nodes in charging cell $q_{i}$
$num_{i}$	Number of sensor nodes in charging cell $q_{i}$
$r_{i}$	Energy consumption rate of sensor node *i*
$re_{i}$	Residual energy of sensor node *i*
$E_{c}^{i}$	Charging amount of sensor node *i*
*E*	Maximum energy of sensor battery
$U_{i}$	Received power of sensor node *i*
$U_{full}$	Transmit power of MC
$re_{mc}$	Residual energy of MC
$E_{mc}$	Maximum energy of MC
*v*	Moving speed of MC
*c*	Energy consumption of MC
$l_{mc}$	Location of MC

**Table 3:** Experiment parameters.

Parameter	Value
*L*, The side length of the square area	1000 m
*D*, Charging range of MC	2.7 m
Number of Nodes	100, 150, 200
$E_{mc}$, MC maximum energy	1000 kJ
*v*, Speed of MC	5 m/s
$U_{full}$, Transmit power of MC	20 w
*c*, Energy consumption of MC	50 J/m
*E*, Sensor maximum energy	6000 J
θ, Threshold factor	0.5
$r_{i}$, Energy consumption of sensor	0.3∼1 J/s
$re_{i}$, Initial Residual Energy of sensor	500∼6000 J
*b*, Mini-batch	32
*B*, Capacity of buffer B	20,000
γ, Discounted factor	0.95
η, Learning rate for gradient descent	$1\times 10^{-6}$
$\epsilon_{k}$, Probability of choosing a random action	[0.01, 1]
*n*, The steps $\epsilon_{k}$ change from 1 to 0.01	100

**Table 4:** Average charging cost and energy efficiency.

Schemes	100 Nodes		150 Nodes		200 Nodes	
	Average Cost (m)	Energy Efficiency (%)	Average Cost (m)	Energy Efficiency (%)	Average Cost (m)	Energy Efficiency (%)
OTM3DQN	138.79	46.09%	130.28	47.19%	132.79	49.57%
RMP-RL	140.00	41.96%	132.82	43.56%	138.91	46.59%
ACRL	142.84	43.13%	137.76	44.30%	141.60	45.75%
Greedy	143.61	45.81%	141.88	44.53%	145.91	45.26%
FCFS	171.77	39.14%	188.31	40.41%	227.37	35.06%

## Data Availability

The data are not publicly available due to it is not permitted.
